# Resilience and Mental Health Among Syrian Refugee Children in Jordan

**DOI:** 10.1007/s10903-021-01180-0

**Published:** 2021-04-26

**Authors:** Rebecca Dehnel, Heyam Dalky, Subashini Sudarsan, Wael K. Al-Delaimy

**Affiliations:** 1 UC San Diego School of Medicine, La Jolla, CA USA; 2 Jordan University of Science and Technology, Irbid, Jordan; 3 UC San Diego, La Jolla, CA USA; 4 Herbert Wertheim School of Public Health, UC San Diego, La Jolla, CA USA

**Keywords:** Syrian refugees, Child and adolescent psychiatry, Trauma, Depression, Resilience

## Abstract

Refugee populations are at high risk of experiencing trauma and developing negative mental health outcomes. The resilience of Syrian refugee children is not well established as far as modifying the association between trauma and mental illness. A total of 339 Syrian refugee children aged 10 to 17 were surveyed to assess resilience, depression and history of trauma. All children reported exposure to at least one traumatic event, 48.6% reported exposure to highly salient traumatic events such as being held hostage, kidnapping or imprisonment. High rates of suicidal ideation and depression symptomatology were found. Resilience was strongly inversely related to depression. Relational support was found to be the most protective resilience factor and was the most highly correlated with less depressive symptomatology. Empowering children and families to build resilience through social support may be a viable prevention and management approach to other unaffordable or unavailable treatments for mental illnesses.

## Introduction

The Syrian refugee crisis is the largest humanitarian emergency of our time according to the United Nations High Commissioner for Refugees. An estimated 5.6 million people have fled Syria since 2011 and 6.6 million are considered internally displaced [1]. Along with fleeing their homes, many Syrian refugees have faced the deaths of those closest to them, survived emotional trauma and physical torture and live with the daily reminder that their family’s future is uncertain. Of the 12 million displaced Syrians it is estimated that half are children under the age of 18 with 40% being under the age of 12 [2]. In addition to Turkey, Lebanon and Iraq, Jordan continues to be one of the most overwhelmed nations impacted by mass Syrian refugee migration [3]. The majority of Syrian refugee families arrived in Jordan in 2013 due to the escalation of violence with thousands more continuing to seek refuge in the years since. In 2018, 80% of Syrian refugee families in Jordan lived within Jordanian communities with an estimated 93% living below the poverty line [1].The cities of Irbid and Ramtha in Northern Jordan are considered highly impacted areas due to this mass migration and their Syrian populations were the focus of this study.

It has been long understood that the refugee experience has a strong psychological effect on children [4, 5]. Throughout multiple contexts, history of adverse childhood events is a well-established risk factor for developing mental health disorders [6–8]. Exposure to violence in particular has been shown to predispose children to developing mental illness such as depression, anxiety and posttraumatic stress disorder (PTSD), all of which are likely to persist throughout adulthood [9–11]. Based on a study of Syrian refugee children living in Islahiye camp in Turkey, 79% had experienced a death in the family, 60% had seen someone being physically assaulted and 30% had themselves been physically hurt [12]. The Bahçeşehir study of Syrian refugee children found that almost half displayed symptoms of PTSD in addition to 44% reporting symptoms of depression [13]. Given this impact, it is crucial to better understand the factors that are correlated with negative and positive mental health outcomes in refugee children and the literature in this regard is limited*.* Refugee population studies [14–19] often focus on maladaptive behaviors and factors that lead to higher prevalence and severity of depression and anxiety, but far fewer [5, 20] have examined the protective factors that may have shielded refugee children from developing more severe mental health outcomes.

Although the psychological effect of refugee experience is substantial, the majority of refugees do not develop significant mental health disorders [21, 22]. Refugee children demonstrate incredible strength by coping with conditions of extreme deprivation and survive adversity which is often under-recognized by the Western medical model. The symptomatic distress of the refugee experience is placed in the realm of psychopathology rather than being viewed as a normal response to an abnormal situation [23, 24]. Shifting toward a strength-based approach focused on resilience may allow clinicians and community-outreach programs to better support refugee children to reinforce and build their own resilience rather than pathologize them.

Resilience can be understood as patterns of positive adaptation in the context of significant risk or adversity [25, 26]. It implies inner strength due to individual characteristics such as optimism, adaptability and perseverance. It also extends beyond the individual and is deeply influenced by social processes that reside in relationships among people, such as family support, as well as contextual factors such as community involvement and faith [27–29]. Some of the earliest work in the field of childhood resilience focused on children at high risk for psychopathology who demonstrated unexpected adaptive behaviors and mindsets [30]. Instead of treating these children as outliers, Garmezy and colleagues focused on what factors made these children uniquely resilient [31]. Werner’s cohort study of at-risk infants in the 1980s found that factors such as connection with family, support systems outside of the home and attributes such as sociability were central to protecting at-risk children from developing poor mental health outcomes [32]. By the turn of the century resilience research rapidly expanded and was examined in many at-risk situations such as physical and emotional abuse [33], catastrophic life events [34], chronic illness [35] and community violence [36]. More recently studies have focused on the importance of examining mental health through the lens of resilience focusing on strength and capacity for growth [26, 37–40].

Studies suggest that a resilience framework can be applied to interventions by focusing on the protective mechanisms that shield individuals from developing mental health disorders. However, the role of resilience varies across cultures and causes of trauma, and what is impactful in one context might not be useful in another [41]. If there is evidence that resilience-based intervention can help prevent or limit mental illness among children, it could create a paradigm shift in addressing mental illness in war-traumatized children in low resource and refugee settings. This study focuses on highly traumatized Syrian children to address the effect of trauma on depression and the role of resilience in this association and its consequences, especially for those who have limited or no access to specialized psychiatric care.

## Method

### Participants and Procedure

Participants for this study (N = 339) were Syrian refugee children and adolescents living in Jordan. Families were approached for participation between the period of July to October 2018 at a major community clinic for refugees in Ramtha, in addition to numerous Syrian community centers in Irbid. Additional families were contacted through community-based organizations in an attempt to create a diverse sample that represents the Syrian refugee communities in Jordan. Even though most Syrian refugee families live within the communities of neighboring countries, most research has focused on families living in refugee camps. In an effort to get a more representative sample, families living within the community were the focus of this study.

Participants were enrolled with their primary guardian. Interested families were eligible if they were Syrian refugees and had a child age 10 to 17 interested in participating. At each location, child interviews were performed in Arabic in a private designated area. Due to illiteracy, the majority of children were verbally administered the questionnaires by trained research assistants. Child participants were given a toy after completing the interview. Research protocol was reviewed and approved by relevant institutional review boards and all study participants completed the informed consent in Arabic verbally due to the sensitive nature of the research questions and the importance of confidentiality in this setting.

### Interviewer Training

Research assistants were Jordanian graduate students with experience working within Syrian refugee communities. Assistants were thoroughly trained in administration of all surveys including a focus on ethics procedures given the vulnerability of the target population and the sensitivity of the questions being posed, in addition to specific instructions on how to provide referrals and support. All children who expressed any form of suicidal ideation were immediately referred and followed-up with by clinic personnel per clinic protocol.

### Instruments

All instruments were translated into Arabic and validated in previous studies or were provided in Arabic by the tool developer [41, 42].

#### Demographics

A demographics questionnaire developed for this project assessed basic demographic information in addition to specific questions about access to mental health care.

#### History of Trauma

Childhood history of trauma was assessed using the Harvard-Uppsala Trauma Questionnaire for Children (HUTQ-C) [43]. This survey was developed from the Harvard Trauma Questionnaire (HTQ) which is a validated cross-cultural survey which measures trauma events and posttraumatic symptomatology in adults [44]. The child version, HUTQ-C, contains 30 traumatic event items which refugee children are most likely to be exposed to and impacted by. The child endorses if they have ever heard about, witnessed or experienced a given item, such as “forced separation from family members”. Individuals with higher scores are characterized as having a higher degree of traumatic experience. Events classified as highly salient included maltreatment/assault, held hostage, kidnapped, imprisoned, forced isolation from others, forced separation from family members, tortured, being close to death, murder of family or friend, and murder of stranger.

#### Depression

Depressive symptomatology was assessed using the Children’s Depression Inventory 2: (CDI-2) [45]. This survey is a brief self-report test that assesses cognitive, affective and behavioral signs of depression in children and adolescents. The CDI-2 contains 28 items, each of which consist of three statements. For each item, the individual selects the statement that best describes their feelings. For example, “I do not feel alone”, “I feel alone many times”, or “I feel alone all the time”. Individuals with higher scores on the CDI-2 are characterized as having higher rates of depression. The first version of the scale was first adapted for use with Arabic speakers in 2006 with an overall alpha reliability coefficient of 0.85 [46]. Multiple cut off scores for significant depression using this measure have been theorized in the literature for various populations. For example, a score of 13 and above suggests a high likelihood of clinical depression [47], while a score of 16 and above indicated a level of depressive symptoms that impacts children’s everyday activities with family, friends and at school [48].

#### Resilience

Resilience was assessed using the 28-item Child and Youth Resilience Measure (CYRM-28) [49, 50]. This full length version of the survey is a 28-item measure of resilience with three subscales: Individual capacities and resources, relationships with primary caregivers and contextual factors. The individual component represents personal and social skills, the relational component reflects the child’s social support, such as the relationship with their primary caregivers, and the contextual component assesses influence of spirituality and environmental influences that contribute to the child’s resilience. Together these areas reflect the notion that resilience is a multifaceted interaction between the individual and their environment [51]. The CYRM asks the participant to select the extent to which statements describe them. For example, “I know what I am good at”. In this version for use with children and adolescents all items are rated on a 3-point Likert scale as, “No”, “Sometimes”, and “Yes”. Higher scores indicate higher resilience attributes. The 28-item CYRM also has a shortened question subset, the CYRM-12 [52]. This 12-item measure contains 12 of the original 28 items and has been reported as a better fit for measuring resilience among refugees [41]. The Arabic version of the CYRM-12 was validated in Jordan [41] and the CYRM-28 was supported for use in this context by other studies [53–56].

## Analysis

### Statistical Methodology

Descriptive statistics were run on all variables pertaining to the analysis. Scores of depression, trauma and resilience were obtained through the summation of answer scores for each questionnaire: CDI-2 for depression, HUTQ-C for trauma, and CYRM-28/CYRM-12 for resilience. All variables were used as continuous variables in the regression analyses as this generates the highest power to detect significant associations in multivariable regression that includes other covariates. The median value was used as a cut-off point to generate categorical variables of depression and resilience since defined cut-off scores for these measures are not established or are highly debated within the literature.

### Effect Modification

We hypothesize that exposure to trauma in childhood will lead to higher prevalence of depression symptomatology and that higher resilience among children will lessen the effect of trauma on depression. To test this hypothesis, effect modification analysis was performed. In order to create more sensitive analyses through stratification, resilience was divided based on the tertile scale of the CYRM-28 creating a “low resilience group” (total CYRM-28 score between 0 to 68), “moderate resilience group” (total CYRM-28 score between 69 and 74) and “high resilience group” (total CYRM-28 score above 74). The relationship between trauma (measured as HUTQ-C score) and depression (measured as CDI-2 score) was then tested using linear regression among the three groups of children that were divided based on the resilience score (Table 1). All of the three variables, depression (CDI-2 score), trauma (HUTQ-C score) and resilience (CYRM-28 score) are all treated as continuous variables.

**Table 1:** Modification effect analysis: Effect of trauma on depression based on resilience tertile

Resilience group (CYRM-28 Score)	Depression ~ Trauma		
	Trauma estimate	p-value	95% CI
Low Resilience (< 69)	0.14	0.03	(0.019, 0.275)
Moderate Resilience (69–74)	0.19	0.02	(0.033, 0.355)
High Resilience (> 74)	1.03	0.13	(−0.029, 0.236)

### Secondary Analysis

Given the reported better fit of the CYRM-12 in refugee populations, the CYRM-12 was used during secondary analysis. The aim of this analysis was to examine if a particular component of resilience had a stronger relationship with depression compared to others. The resilience score using the CYRM-12 scale was regressed on depression. The CYRM-12 was then divided into its three established resilience subcategories: contextual, relational and individual (Table 2). Each of three subcategories of the CYRM-12 were then regressed with depression to examine the strength and magnitude of the relationship. CDI-2 and CYRM-12 scores were treated as continuous variables.

**Table 2:** CYRM-12 scale with subcategories

Question #	Question
*Individual:*	
8	I try to finish what I start
15	I know where to go in my community to get help
21	I am aware of my strengths
25	I have opportunities to develop skills that will be useful later in life
*Relational:*	
5	My parents/caregiver(s) watch me closely
6	My parents/caregiver(s) know a lot about me
17	My family stands by me in difficult times
24	I feel safe when I am with my family/caregiver(s)
*Contextual:*	
3	Getting an education is important to me
9	Spiritual beliefs are a source of strength for me
22	I participate in organized religious activities
27	I enjoy my community traditions

### Statistical Software

All analysis was performed using the R statistical package (Version: Rstudio 1.4.156). Effect modification analysis was run using the mediate package in R [57].

## Results

### Demographic Characteristics

A total of 339 adults and 339 children participated. Adults included the child’s mother, father or primary caregiver. All child participants were Syrian refugees with most endorsing being Arab (98.8%), with a mean age of 13.4 years (range 10 to 17), and a mean educational grade completion of 7.2 (range 0 to 12). Most child participants were female (74.3%) and many children (40.7%) had parents who described their family income as poorly taking care of their needs. These findings are summarized in Table 3.

**Table 3:** Participant demographics and variable descriptives

Demographics	
Age in years	
Mean (SD)	13.4 (2.3)
Median (Range)	13 (10–17)
Gender, n (%)	
Male	85 (25.1)
Female	252 (74.3)
Unreported	2 (0.01)
Ethnicity, n (%)	
Arab	335 (98.8)
Other	1 (0.003)
Unknown	3 (0.01)
Guardian completing form, n (%)	
Mother	311 (91.7)
Father	18 (5.3)
Grandmother	4 (1.2)
Grandfather	2 (0.6)
Other	4 (1.2)
Family income taking care of needs, n (%)	
Poorly	138 (40.7)
Psychosocial variables	
Child depression (CDI-2)	
Mean (SD)	14.4 (7.8)
Median (Range)	14 (0–40)
Child Trauma (HUTQ-C)	
Mean (SD)	8.8 (9.4)
Median (Range)	6 (0–57)
Exposed to Trauma, n (%)	339 (100)
Exposed to highly salient Trauma, n (%)	165 (48.6)
Child resilience (CYRM-28)	
Mean (SD)	70.9 (8.5)
Median (Range)	72 (0–84)
Child resilience (CYRM-12)	
Mean (SD)	31.1 (4.1)
Median (Range)	32 (0–36)

### Trauma Exposure

**Fig. 1:**
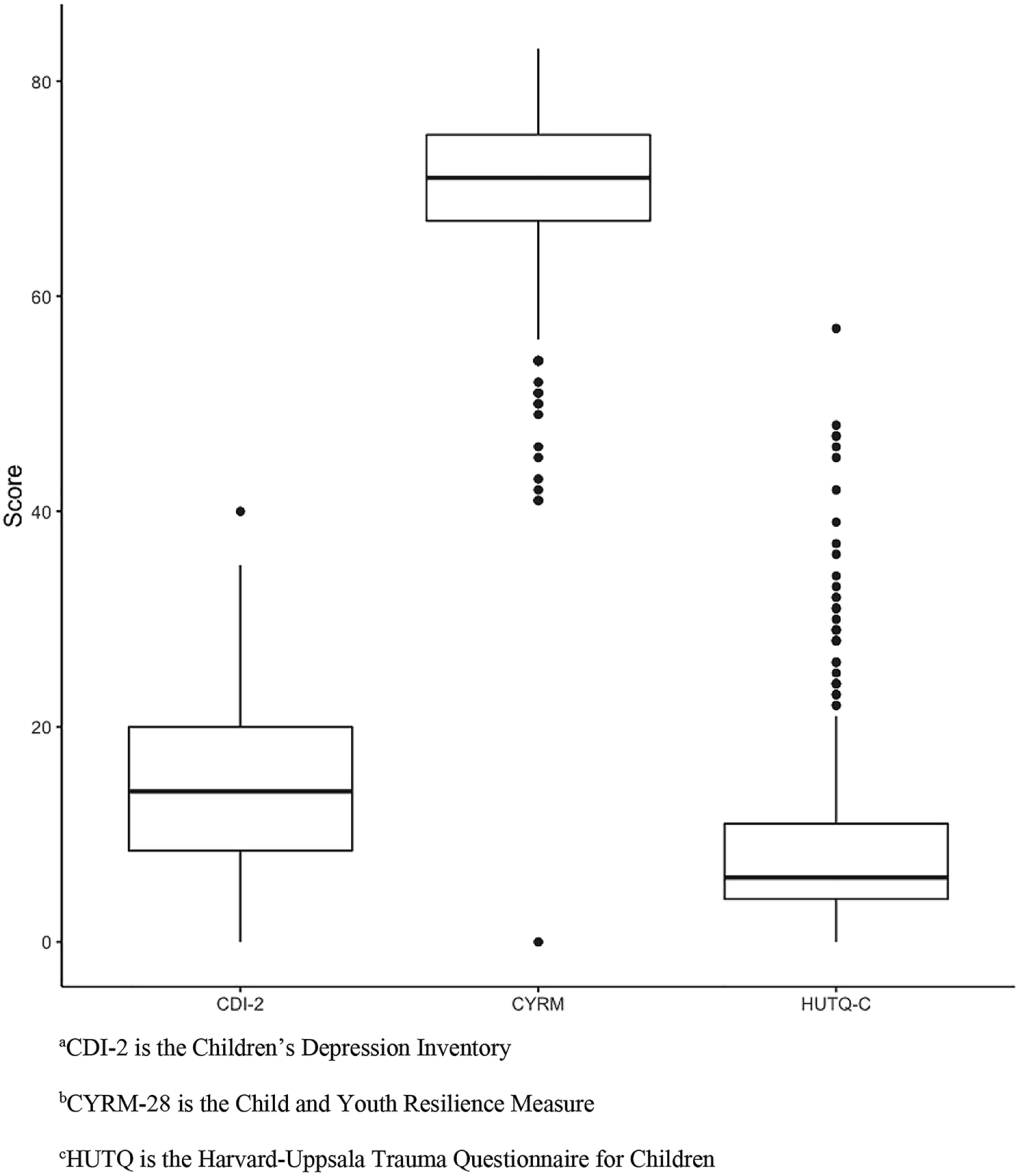
Whisker Box Plot of Depression Score (CDI-2)^a^, Resilience Score (CYRM-28)^b^ and Trauma Score (HUTQ)^c^. ^a^CDI-2 is the Children’s Depression Inventory, ^b^CYRM-28 is the Child and Youth Resilience Measure, ^c^HUTQ is the Harvard-Uppsala Trauma Questionnaire for Children

The mean score on the HUTQ-C was 8.8 with a minimum score of 0 and a maximum score of 57 as seen in Fig. 1. The most common traumatic life events reported included “car accidents”, “robberies” and “war experience”. All children (100%) endorsed exposure to at least 1 traumatic event. A slight majority of children (51.3%) reported being exposed to traumatic events with a score greater than 5 on the HUTQ-C. In addition, nearly half of children (48.6%) endorsed hearing about, witnessing or experiencing a highly-salient traumatic event. These findings are summarized in Table 3. Table 4 lists all of the heard, witnessed and experienced traumas reported in this sample of children.

**Table 4:** Reported percent of traumatic life events (n = 339) among children

Trauma event	Heard about n (%)	Witnessed or experienced n (%)
Road accident	120 (35.4)	109 (32.2)
Accident at school	54 (15.9)	82 (24.2)
Accident in spare time	33 (9.7)	38 (11.2)
Technical accident	31 (9.1)	25 (7.4)
Natural disaster	41 (12.1)	18 (5.3)
Serious injury	38 (11.2)	53 (15.6)
Robbery	74 (21.8)	27 (8.0)
Maltreatment/assault	37 (10.9)	32 (9.4)
Mobbing	34 (10.0)	41 (12.1)
Hostage	25 (7.4)	8 (2.4)
Kidnapping	58 (17.7)	10 (2.9)
Imprisonment	51 (15.0)	19 (5.6)
Lost/disappeared	49 (14.5)	23 (6.8)
Forced isolation from others	24 (7.1)	10 (2.9)
Forced separation from family members	22 (6.5)	8 (2.4)
Lack of food or water	32 (9.4)	21 (6.2)
Lack of shelter	23 (6.8)	45 (13.3)
War experience	105 (31.0)	234 (69.0)
Family member mobilized to war	23 (6.8)	27 (8.0)
Torture	41 (12.1)	11 (3.2)
Being close to death	24 (7.1)	38 (11.2)
Forced medical care	16 (4.7)	11 (3.2)
Terrifying hospital experience	17 (5.0)	18 (5.3)
Ill with no access to treatment	21 (6.2)	26 (7.7)
Murder of family or friend	55 (16.2)	41 (12.1)
Murder of stranger	48 (14.2)	20 (5.9)
Unnatural death of family or friend	21 (6.2)	12 (3.5)
Brainwashing	15 (4.4)	3 (0.9)

### Depression Scores

The mean score on the CDI-2 was 14.4 with a minimum score of 0 and a maximum score of 40 as seen in Fig. 1. Using either the cut-off score of 13 and 16 yielded significantly high rates of depression within this sample. A slight majority of children (51.8%) met criteria for having significant depressive symptoms as outlined by Kovac’s cutoff of a score of 13 and 40.4% met the cut off score of 16 as outlined by Roelofs [47, 48]. It should also be noted that 27.8% of children endorsed suicidal ideation. There were 81 children (23.8%) endorsed the statement “I think about killing myself, but would not do it” and 13 children (4%) endorsed the statement “I want to kill myself”. These findings are summarized in Table 3.

### Resilience Scores

The mean score on the CYRM-28 was 70.9 with a minimum score of 0 and a maximum score of 84 as seen in Fig. 1. When examining the resilience subscales of the CYRM-28, the “Individual” mean score was 26.6 (SD = 3.7) out of 33 possible points suggesting that 80.6% of the children had individual resilience characteristics such as personal skills, peer support and/or social skills. The “Relational” mean score was 18 (SD = 2.6) out of 21 possible points suggesting that 85.6% of the children had relational resilience characteristics such as physical and/or psychological caregiving by their caregivers. The “Contextual” mean score was 26.3 (SD = 3.3) out of 30 possible points suggesting that 87.6% of the sample had contextual resilience characteristics such as spirituality, education and/or culture. These findings are summarized in Table 3.

### Correlation Between Variables

Kendall’s correlation coefficients were calculated to assess the relationship between the variables. There was a significant negative correlation between resilience (CYRM-28) and depression (CDI-2) scores (−0.33, *p* < 0.001). In addition, there was a statistically significant positive but weak correlation between traumatic life events (HUTQ-C) and depression (CDI-2) (*r* = 0.1, *p* = 0.02). These correlations suggest that higher levels of resilience resulted in lower levels of self-reported depression symptoms and that more exposure to trauma and higher levels of self-reported depression symptoms are related. However, traumatic life events were not significantly correlated with resilience.

### Effect Modification

Resilience appeared to have a positive effect on blunting the impacts of trauma on depression in this population. There was a significant association between trauma and depression among those with the lower and moderate resilience. Among children who were categorized as having low resilience, the relationship between depression and trauma was significant, but had a low estimate of trauma (0.15, p-value = 0.026). A similar result was also observed among children who were considered to have moderate resilience (trauma estimate = 0.19, p-value = 0.02). The high resilience group showed no significant relationship between trauma and depression.

### Secondary Analysis

All three subcategories of the CYRM-12 were significantly associated with depression with a negative correlation (i.e. as the resilience score increased depression score decreased) as seen in Table 5. The magnitude of individual and contextual components of resilience was 0.71 and the relational component was 1. Overall, the relational component of resilience had the strongest effect on depression.

**Table 5:** Secondary analysis: effect of resilience on depression based on resilience subcategory

Resilience subcategories (CYRM-12 Score)	Depression ~ Trauma	
	Resilience estimate	p-value
Individual	−0.71	< 0.001
Relational	−1.04	< 0.001
Contextual	−0.71	< 0.001

## Discussion

It has long been understood that the forced displacement and trauma associated with the refugee experience have significant impacts on children. Now nearly a decade since the start of the Syrian War and resulting refugee crisis we are still navigating these impacts and developing strategies to better support Syrian refugee children.

Consistent with prior studies, this cohort of Syrian refugee children endorsed very high rates of depression even after using multiple suggested cut-off scores. The use of the CDI-2 was not intended to be diagnostic, but rather to broadly assess the depressive symptomatology and the prevalence was high. There is significant disagreement within the literature about the validity of CDI as a diagnostic measure [58], however, using either cut-off score of 13 or 16 in this sample yielded significantly high rates of depressive symptomatology with 55.4% representing clinical depression (reflected by cut-off score of 13) and 40.4% having depressive symptomatology likely interfering with children’s everyday activities with family, friends and at school (reflected by cut-off score of 16). This is consistent with prevalence rates found in other work such as a meta-analysis involving 17 studies and 7920 refugee children which found that 53% of children expressed significant symptoms of depression [18]. Additionally, the surprisingly common endorsement of suicidal ideation by children further supports that the rates of depressive symptomatology found are substantial. Nearly a third of children endorsed a form of suicidality, which was a very unexpected finding given the especially strong stigma against suicide in Muslim culture [59] giving painful insight into the reality of these children. Better understanding of how to assess suicidality among refugee children and how to manage it will be important next steps.

In addition to the high prevalence of depression, it was also found that all Syrian refugee children in this group had been exposed to a form of trauma with nearly half endorsing exposure to at least one highly salient traumatic event. As expected, there was a significant relationship between history of trauma and depression, but the correlation was not as strong as in prior work. However when severe trauma was used, there was a stronger association. This weaker association could reflect the measure not accurately capturing childhood trauma history. When interviewed, children frequently did not endorse exposure to all of the traumatic events that their primary caregiver stated they had been exposed to. One might suggest that if a child does not recall or endorse exposure to a traumatic event then it may not be playing as significant of a role in their psychological well-being, but the impact of trauma on child development and mental health is arguably far more complicated. Future studies should consider using measures with a child and caregiver component to assess exposure to trauma more accurately. While the impact score was less than expected, the relationship between trauma and depression was significant which supports the long-held understanding that exposure to trauma in childhood predisposes children to experience symptoms of depression [10]. The current sample was not followed up for progression of symptoms, but this population is at high risk of having persistence of these symptoms throughout their lives, hindering their readjustment into their new communities and ultimately developing into negative mental health outcomes.

Many prior studies have examined the effects of resilience in childhood [60], but this is the first study to our knowledge that has examined the role of resilience in protecting refugee children exposed to traumatic events from symptoms of depression. The mean resilience score within this Syrian refugee population was within a half of a standard deviation of European and Indian child population means studied using this measure with the same scale [61]. As hypothesized, resilience was found to serve as a strong protective factor against depression and no significant correlation was found between history of trauma and resilience. A lack of correlation between trauma and resilience was also found in the Panter-Brick study of Syrian refugee children [41], but the explanation for these findings is still unclear. History of trauma and resilience being independent factors in addition to Syrian refugee children having resilience ratings comparable to study populations of European and Indian children could suggest that resilience is an innate characteristic of each individual child and that the experiences of trauma do not bear weight on their resilience. More likely however, the relationship between trauma and resilience is multifaceted and the surveys that were used did not capture the intricacies. Trauma may embolden the resilience of some children and compromise the resilience of others. This is a difficult relationship to understand without pre-exposure data characterizing who these children were before their exposure to trauma and how it shaped them and their resilience.

From what we can characterize based on the secondary analysis examining the subcategories of resilience, our findings suggest that relational resilience (i.e. the degree to which a child feels social support) had the most significant effect on the likelihood of endorsing symptoms of depression. This is an inspiring finding because perceived social support, unlike individual resilience traits, is modifiable and could serve as a very meaningful focus for future community interventions. Supporting this argument, Southwick states that resilience extends beyond the individual and is embedded in larger social systems through a multitude of facets [62]. Any of the numerous social support facets could be the focus of resilience interventions. If done in a community setting by partnering with parents these approaches could have a substantial effect on childhood resilience and ultimately improve existing depressive symptomatology and mitigate the likelihood of developing new or worsening depression. However, this would require a more specific examination of what social support factors most strongly correlated with positive mental health outcomes in Syrian refugee children and a modifiable intervention strategy given that the social support needs of one child or one community may be completely different than another.

The results of the present study should be interpreted keeping the following limitations in mind. We were unable to follow participants longitudinally to document the association of resilience and depression over time. We are also dependent on the recall of children regarding trauma and depression symptoms and this subjective response might underestimate the true associations found as there was no pediatric psychiatrist to formally assess for clinical depression. However, most studies have relied on these measures for children and the instruments have been validated previously. This is also not a random representative sample of Syrian children, however we believe this would not have a major effect on the conclusions found, given there is consistency between the high percentages of depression and resilience in our study compared to others. Additionally, there were no Syrian children who were not exposed to war trauma included for comparison purposes, which could explain the limited association between history of trauma and depression. Lastly, the majority of children in this sample were female which may have had unintended influence on the results given gender differences found in mental health research. This is likely the byproduct of female children being more often present in family homes or with their parents in clinics when surveys are collected in these settings. The influence of gender roles on mental health within this population is an important one and the impact of female children spending more time with their parents than their male counterparts is an interesting future direction for this work given the impact of social support on mental health.

In summary, the current study found a statistically significant positive correlation between trauma and depression, no relationship between trauma and resilience and a strong negative correlation between resilience and depression. Resilience was significantly correlated with less depression symptomatology and appears to be a strong protective factor, but its influence on depression in the context of trauma and the strategy and effectiveness of promoting resilience in children through interventions remains unclear. Likewise, low resilience in childhood appears to be a strong predictor of having depressive symptoms and could theoretically be used to predict which groups of children are at the highest risk of having poor mental health outcomes in the future without strong resilience to protect them.

The Syrian state’s collapse amidst civil war will take decades to recover and there is now an entire generation of Syrian children whose growth and development will forever be influenced by the traumas of their childhood. A better understanding of factors such as resilience and history of trauma will better inform our ability to assess the risk of poor mental health outcomes in these children and optimize outreach efforts in these communities.
